# Accelerated stress CMR for the detection of significant coronary artery disease: a prospective randomized diagnostic accuracy study

**DOI:** 10.1093/ehjci/jeaf322

**Published:** 2025-11-20

**Authors:** Mohamed Elshibly, Simran Shergill, Anju Velvet, Aisha Gohar, Shreyansh Pattani, Kelly S Parke, Rachel England, Aida Moafi, George Hudson, Jeffrey Khoo, Declan P O’Regan, Olivia Wu, David Adlam, Peter Kellman, Alasdair McIntosh, Alex McConnachie, Andrew Ladwiniec, Gerry P McCann, J Ranjit Arnold

**Affiliations:** Department of Cardiovascular Sciences, University of Leicester, The National Institute for Health and Care Research Leicester Biomedical Research Centre and British Heart Foundation Centre of Research Excellence, Glenfield Hospital, Groby Road, Leicester, LE3 9QP, UK; Department of Cardiovascular Sciences, University of Leicester, The National Institute for Health and Care Research Leicester Biomedical Research Centre and British Heart Foundation Centre of Research Excellence, Glenfield Hospital, Groby Road, Leicester, LE3 9QP, UK; Department of Cardiovascular Sciences, University of Leicester, The National Institute for Health and Care Research Leicester Biomedical Research Centre and British Heart Foundation Centre of Research Excellence, Glenfield Hospital, Groby Road, Leicester, LE3 9QP, UK; Department of Cardiology, University Hospitals of Leicester NHS Trust, Leicester, UK; Department of Cardiology, University Hospitals of Leicester NHS Trust, Leicester, UK; Department of Cardiovascular Sciences, University of Leicester, The National Institute for Health and Care Research Leicester Biomedical Research Centre and British Heart Foundation Centre of Research Excellence, Glenfield Hospital, Groby Road, Leicester, LE3 9QP, UK; Department of Cardiovascular Sciences, University of Leicester, The National Institute for Health and Care Research Leicester Biomedical Research Centre and British Heart Foundation Centre of Research Excellence, Glenfield Hospital, Groby Road, Leicester, LE3 9QP, UK; Department of Cardiovascular Sciences, University of Leicester, The National Institute for Health and Care Research Leicester Biomedical Research Centre and British Heart Foundation Centre of Research Excellence, Glenfield Hospital, Groby Road, Leicester, LE3 9QP, UK; Department of Cardiovascular Sciences, University of Leicester, The National Institute for Health and Care Research Leicester Biomedical Research Centre and British Heart Foundation Centre of Research Excellence, Glenfield Hospital, Groby Road, Leicester, LE3 9QP, UK; Department of Cardiovascular Sciences, University of Leicester, The National Institute for Health and Care Research Leicester Biomedical Research Centre and British Heart Foundation Centre of Research Excellence, Glenfield Hospital, Groby Road, Leicester, LE3 9QP, UK; Department of Cardiology, University Hospitals of Leicester NHS Trust, Leicester, UK; Medical Research Council Laboratory of Medical Sciences, Imperial College London, London, UK; Health Economics and Health Technology Assessment, School of Health and Wellbeing, University of Glasgow, Glasgow, UK; Department of Cardiovascular Sciences, University of Leicester, The National Institute for Health and Care Research Leicester Biomedical Research Centre and British Heart Foundation Centre of Research Excellence, Glenfield Hospital, Groby Road, Leicester, LE3 9QP, UK; Department of Health and Human Services, National Heart, Lung, and Blood Institute, National Institutes of Health, Bethesda, MD, USA; School of Health and Wellbeing, Robertson Centre for Biostatistics, University of Glasgow, Glasgow, UK; School of Health and Wellbeing, Robertson Centre for Biostatistics, University of Glasgow, Glasgow, UK; Department of Cardiovascular Sciences, University of Leicester, The National Institute for Health and Care Research Leicester Biomedical Research Centre and British Heart Foundation Centre of Research Excellence, Glenfield Hospital, Groby Road, Leicester, LE3 9QP, UK; Department of Cardiology, University Hospitals of Leicester NHS Trust, Leicester, UK; Department of Cardiovascular Sciences, University of Leicester, The National Institute for Health and Care Research Leicester Biomedical Research Centre and British Heart Foundation Centre of Research Excellence, Glenfield Hospital, Groby Road, Leicester, LE3 9QP, UK; Department of Cardiovascular Sciences, University of Leicester, The National Institute for Health and Care Research Leicester Biomedical Research Centre and British Heart Foundation Centre of Research Excellence, Glenfield Hospital, Groby Road, Leicester, LE3 9QP, UK

**Keywords:** Myocardial perfusion, Stable angina, Fractional flow reserve, Stress perfusion cardiovascular magnetic resonance, Rapid protocols

## Abstract

**Aims:**

In patients with suspected coronary artery disease (CAD), the role of adenosine-stress cardiovascular magnetic resonance (CMR) is well established. However, to meet increasing demand, improving its time efficiency and cost-effectiveness is critical. Recent advances in accelerated, free-breathing cine and scar imaging now enable accelerated stress–perfusion protocols. This study evaluated whether an accelerated, stress-only perfusion protocol achieves non-inferior diagnostic accuracy compared with a standard stress–rest perfusion CMR protocol for detecting significant CAD.

**Methods and results:**

Patients with suspected angina referred for invasive coronary angiography (ICA) underwent two 3-Tesla CMR scans (standard and accelerated protocols), on separate days in randomized order. Significant CAD was defined as fractional flow reserve (FFR) ≤ 0.80 in epicardial vessels ≥2 mm diameter (or quantitative flow ratio ≤0.80 if FFR unavailable). CMR images were evaluated qualitatively with (i) primary per-vessel analysis (determined by two independent readers) and (ii) secondary per-patient analysis (following consensus read). Of 167 prospectively recruited patients, 150 completed both CMR protocols and ICA (mean age 66 ± 10 years, 71% male, CAD prevalence 51%). The accelerated scan was better tolerated by patients, with scan duration 19 ± 5 min (24 min shorter than the standard protocol [95% CI: 23, 25], *P* < 0.001). Compared with standard CMR, accelerated CMR achieved non-inferior per-vessel diagnostic accuracy at a pre-specified 5% non-inferiority margin (+0.7% [−2.7%, 4.0%], *p*_non-inferiority_ = 0.001 and +3.4% [−0.1%, 6.8%], *p*_non-inferiority_ < 0.001 for the two readers). Accelerated CMR also achieved comparable per-patient accuracy (+4.6% [−1.5%, 11.0%], *P* = 0.189 for consensus read; accuracy 88.6%, sensitivity 84.2%, and specificity 93.2%).

**Conclusion:**

Compared to standard stress–perfusion CMR, an accelerated stress–perfusion protocol achieves non-inferior diagnostic accuracy at the vessel level, with a time saving of over 20 min per scan. Accelerated imaging may prove effective in the clinical arena to evaluate patients with suspected angina.

## Graphical Abstract

**Graphical Abstract:**
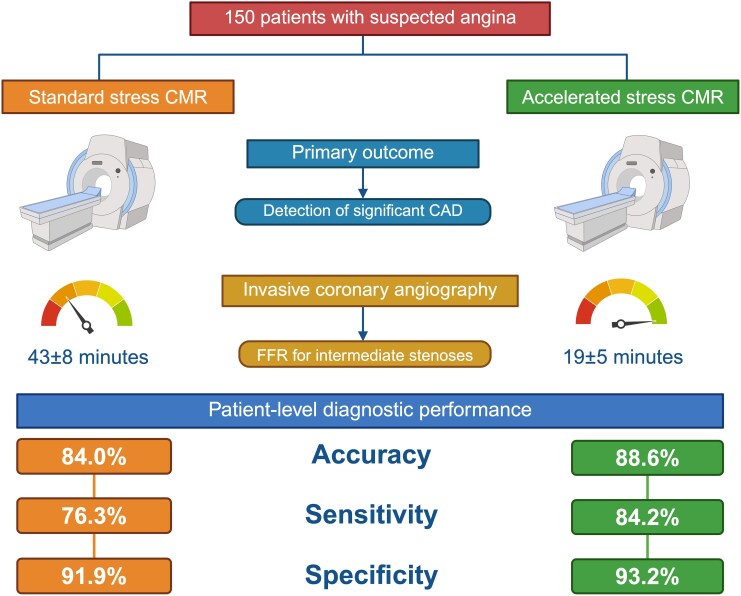

**See the editorial comment for this article ‘Stress CMR in the fast lane: implications beyond image quality’, by E. Nagel and V.O. Puntmann, https://doi.org/10.1093/ehjci/jeag013.**

## Introduction

Coronary artery disease (CAD) remains a leading cause of death and disability worldwide, presenting a critical health and economic burden.^1,2^ In patients with suspected CAD, stress–perfusion cardiovascular magnetic resonance (CMR) serves as an accurate, non-invasive diagnostic test and gatekeeper to invasive coronary angiography (ICA).^3–8^ In patients with known CAD, CMR helps guide treatment decisions and has a well-established prognostic capability.^8–10^

However, a standard stress–perfusion CMR protocol is time-intensive (∼45–50 min, requiring a 60-min time slot) and may prove problematic for patients who are unable to lie flat for prolonged periods or perform repeated breath-holds. Furthermore, regional disparities in access to CMR may hinder its widespread adoption.^11,12^ Hence, there is a strong impetus to develop shortened CMR protocols which can be applied within existing infrastructure. As well as increasing scan tolerability, this will increase patient throughput, with a view to improving efficiency, cost-effectiveness, and accessibility. Capitalizing on developments in accelerated, free-breathing functional cine, and scar imaging with advanced motion correction algorithms, accelerated stress–perfusion protocols are now achievable. Although feasibility has been demonstrated, it remains to be established whether such approaches maintain diagnostic performance and reliability.^13,14^

In this prospective randomized clinical study, we sought to compare the diagnostic accuracy of an accelerated stress-only perfusion protocol with standard stress–rest perfusion CMR in the detection of significant CAD, using invasive fractional flow reserve (FFR) as the reference standard.

## Methods

### Study design

**Figure 1:**
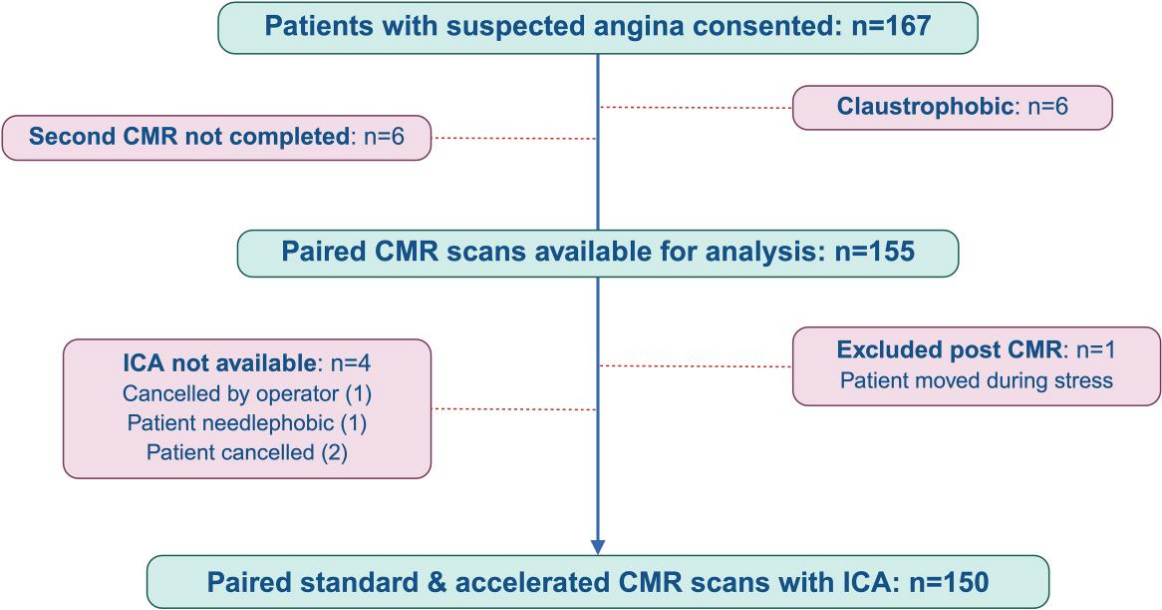
Study flow diagram. Abbreviations: CMR, cardiovascular magnetic resonance; ICA, invasive coronary angiography.

One hundred and sixty-seven consecutive adult patients with *de novo* suspected angina referred for clinically indicated diagnostic ICA were prospectively recruited from a single tertiary cardiac centre (Glenfield Hospital, Leicester, UK) between September 2021 and June 2024 (*Figure 1*). Prior to ICA, patients underwent accelerated and standard stress–perfusion research CMR protocols on the same scanner on different days in randomized order. Exclusion criteria were recent myocardial infarction (≤6 months), unstable angina, previous coronary artery bypass grafting or percutaneous coronary intervention, contraindications to adenosine (second/third-degree atrioventricular block, severe chronic obstructive pulmonary disease, moderate-severe asthma), severe renal dysfunction (estimated glomerular filtration rate <30 mL/min/1.73m^2^), severe claustrophobia, and absolute contraindications to CMR (non-conditional cardiac implantable electronic device, pregnancy, ferromagnetic implants/foreign bodies). The study was approved by the UK National Research Ethics Service (REC reference 19/EM/0295) and registered on ClinicalTrials.gov (NCT05221762). The study was conducted in accordance with the Declaration of Helsinki, and all participants gave written informed consent prior to participation. The study is reported in accordance with the Standards for Reporting Diagnostic Accuracy Studies 2015 guidelines.^15^

### CMR

All CMR scans were performed at 3-Tesla (MAGNETOM Vida [71%] or Skyra [29%], Siemens Healthineers, Erlangen, Germany) with ECG gating, an 18-channel phased array cardiac receiver coil, and 32-channel spine coil. Participants were advised to abstain from caffeine-containing products for at least 12 h prior to vasodilator stress, but routine anti-anginal medications were continued. Hyperaemia was induced with adenosine at a rate of 140μg/kg/min for 3–5 min. Subjects were monitored for symptoms throughout the infusion, with dose escalations at 2-min intervals to 170–210μg/kg/min if there was an insufficient symptomatic and/or haemodynamic response (heart rate increase ≥10 beats/min).^16^ Scan duration was recorded as the time from initiation of the first localizer image to completion of the final sequence. After each scan, participants were asked to complete a self-administered questionnaire on overall experience, comfort, and perceived scan duration.

### Standard CMR protocol

**Figure 2:**
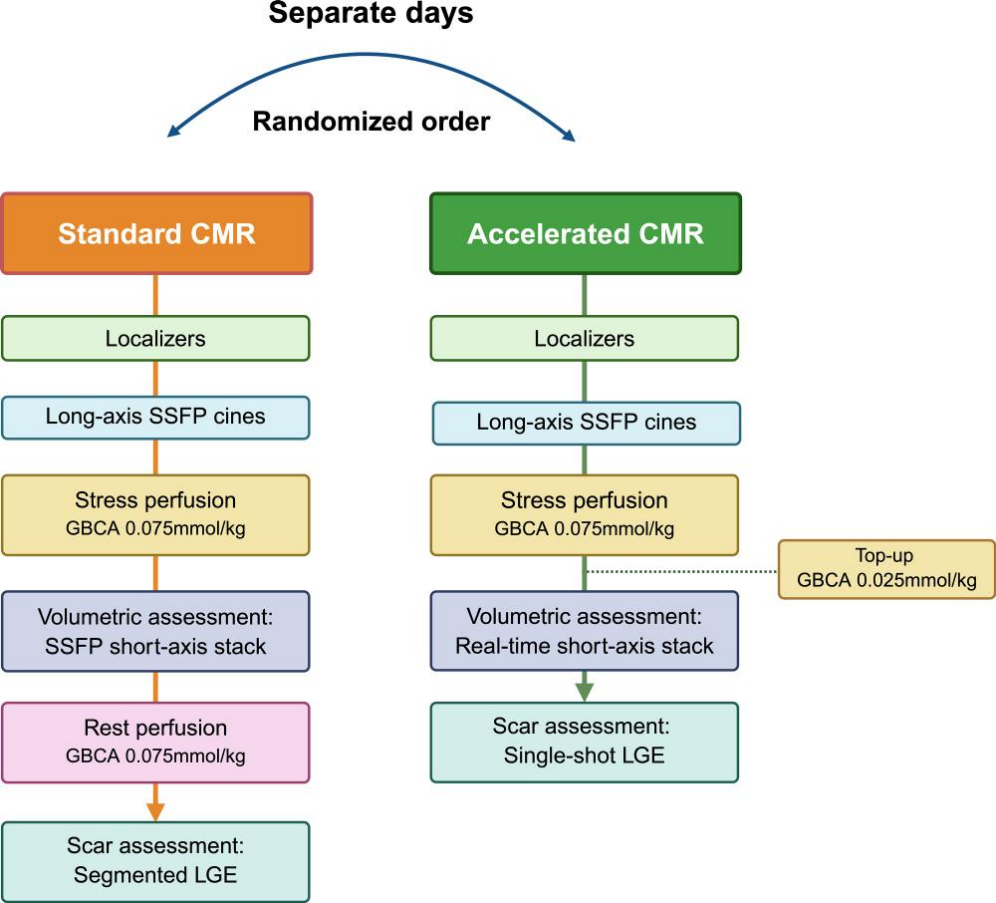
Standard and accelerated CMR protocols. Abbreviations: GBCA, gadolinium-based contrast agent; LGE, late gadolinium enhancement; SSFP, steady-state free precession.

The standard protocol has been previously described in detail (*Figure 2*).^17^ In brief, functional cine imaging was performed in the three long-axis planes (4, 2, 3-chamber) and a contiguous short-axis stack to cover the ventricles using a breath-hold, steady-state free precession pulse sequence (typical sequence parameters: echo time [TE] 1.49 ms, repetition time [TR] 47.5 ms, slice thickness 8 mm, distance factor 25%, matrix 256 × 166, field of view [FOV] 360–400 mm, FOV phase 81.3%, flip angle 80°). A dual-sequence T1-weighted saturation-recovery gradient echo sequence was acquired over 60 heartbeats at rest and during vasodilator stress (typical sequence parameters: TE 1 ms, TR 146 ms, slice thickness 8 mm, matrix 192 × 111, FOV 360–400 mm, FOV phase 75%, flip angle 14°) at three short-axis levels (basal, mid-ventricular and apical), with injection of 0.075 mmol/kg gadoterate meglumine (Dotarem, Guerbet, France or Clariscan, GE HealthCare, Illinois, USA) for each perfusion scan (total dose 0.15 mmol/kg) at 4 mL/s, followed by a 20 mL 0.9% saline bolus.^18^ Late gadolinium enhancement (LGE) imaging was acquired using a breath-hold, T1-weighted segmented inversion recovery gradient echo sequence in the same long and short-axis slice prescriptions as the cine imaging (typical sequence parameters: TE 1.89 ms, slice thickness 8 mm, distance factor 25%, matrix 256 × 152, FOV 360–420 mm, FOV phase 80.5%, and flip angle 20°), with the optimal inversion time determined from a Look-Locker sequence.

### Accelerated CMR protocol

The accelerated protocol consisted of (*Figure 2*):

1. Two-chamber long-axis cine acquired from the axial pilot images, followed by a short-axis localizer to allow acquisition of the four- and three-chamber long-axis cines utilizing a breath-hold, steady-state free precession pulse sequence.
2. Stress perfusion at the basal, mid-ventricular and apical levels of the left ventricle, using the same dual-sequence T1-weighted saturation-recovery gradient echo sequence, with injection of 0.075 mmol/kg gadoterate meglumine at 4 mL/s followed by a 20 mL 0.9% saline bolus.
3. Short-axis cine stack covering the ventricles from base to apex utilizing a free-breathing, ECG-triggered, retro-gated, real-time cine sequence, typically taking 90 s to acquire in our practice (typical sequence parameters: TE 1.09 ms, TR 42.8 ms, slice thickness 8 mm, distance factor 25%, matrix 160 × 92, FOV 360–400 mm, FOV phase 75%, flip angle 45°).^19^
4. Contrast enhanced scar imaging using a non-breath-hold, single-shot, T1-weighted gradient echo sequence (magnitude and phase-sensitive inversion recovery) following 0.025 mmol/kg contrast top-up (administered immediately after stress perfusion, i.e. 0.075 mmol/kg + 0.025 mmol/kg to give a total dose of 0.1 mmol/kg), with matched slice prescriptions to the long- and short-axis cine imaging (typical sequence parameters: TE 1.17 ms, slice thickness 8 mm, distance factor 25%, matrix 224 × 148, FOV 340–380 mm, FOV phase 81.3%, flip angle 40°). A complete set of long- and short-axis LGE images was acquired within ∼60–90 s in our practice. A Look-Locker sequence was used to determine the optimal inversion time from nulling of non-infarcted myocardium, with LGE imaging typically commencing 4 min after contrast-top up injection.

### Image analysis

CMR images were analysed offline, blinded to all participants and angiographic details in a randomized order using certified software (cvi42, Circle Cardiovascular Imaging, Calgary, Canada). Image quality was graded on a four-point scale: 3 = excellent, 2 = good, 1 = moderate, and 0 = unanalysable. Volumetric analysis was quantified with methods as previously described.^17^ Qualitative interpretation of CMR exams was performed by two experienced level 3 CMR accredited observers (each with >15 years of experience) with assessment of resting wall motion, LGE, and perfusion using the 16-segment American Heart Association model. Myocardial segments were further subdivided into subepicardial and subendocardial layers for perfusion and LGE assessment (32-segment model) and ascribed a coronary territory according to standard criteria.^20^ For resting wall motion assessment, standard segmental scoring was performed: 1 = normal, 2 = hypokinesia, 3 = akinesia, 4 = dyskinesia, and 5 = aneurysmal.^21^ For qualitative LGE assessment, scar patterns were graded at a segmental level: 0 = normal, 1 = subendocardial, 2 = transmural, 3 =non-ischaemic, and 4 = insertion point fibrosis. For quantitative assessment of infarction, the full width at half maximum technique was used.^22^ For visual qualitative perfusion assessment, standard segmental scoring was applied: 0 = normal, 1 = subendocardial defect, 2 = transmural defect, and 3 = dark rim artefact. For the standard scan, stress and rest perfusion sequences were displayed simultaneously, whereas for the accelerated scan, only stress perfusion sequences were analysed. Ischaemia was defined as a stress-induced defect in two adjacent segments of a 32-segment model, more extensive than either resting perfusion defect or infarction on matched LGE imaging.^23^ Significant CAD was determined on per-vessel (by two independent readers) and per-patient (following consensus read) levels, defined by the presence of ischaemia and/or infarction. Differences at the patient level were resolved by consensus, and in cases of non-resolved consensus, a third reader adjudicated. In cases of a circumferential perfusion defect not considered to be secondary to epicardial CAD, a diagnosis of probable coronary microvascular dysfunction was subjectively determined, with consideration of the LGE and resting wall motion analysis. Given the absence of a consistently reliable method for dark-rim artefact discrimination, its presence was left to reader judgment, with no definitive criteria applied. However, artefacts were considered if they were short-lived, appeared early with contrast arrival, and did not conform to a specific coronary territory.

### ICA protocol and analysis

Clinically indicated ICA was performed according to standard clinical protocols by interventional cardiologists blinded to imaging findings. FFR was performed in epicardial vessels with visually determined stenosis of 40–90% using an intracoronary pressure wire (PressureWire X, Abbott Vascular, Illinois, USA) and 6-French guide catheter. Hyperaemia was induced with intravenous adenosine, and FFR was calculated as the ratio of mean distal coronary pressure to mean aortic pressure, adjusted for pressure drift.^24^ Significant CAD was defined as FFR ≤0.80 in an epicardial vessel ≥2 mm in diameter. In vessels deemed not safe to perform FFR (subtotal or complete occlusions), the vessel was assumed to have FFR 0.50.^23^ In remaining vessels in which FFR had not been determined and with visually determined ≥25% (mild) stenosis, quantitative flow ratio (QFR) computation was performed offline by an independent observer blinded to imaging and clinical details using QFR v2.2 software (Medis Medical Imaging, Leiden, the Netherlands) with methods as previously described.^17^ QFR ≤0.80 was considered significant.^7^

### Statistical analysis

Statistical analyses were conducted by an independent Clinical Trials Unit at the University of Glasgow. Continuous data are expressed as mean ± standard deviation if normally distributed or median [quartile (Q)1–Q3] if otherwise. Categorical data are presented as absolute values (%). Paired sample *t*-tests and McNemar’s tests were used to compare within-group differences where appropriate. Kappa coefficient was used to assess interobserver agreement. For the primary per-vessel analysis, a non-inferiority test was performed with a pre-specified margin of 5%. The Newcombe–Wilson score method with continuity correction was used to obtain non-inferiority *P*-values and compare the diagnostic accuracy of both protocols by calculating a two-sided 95% confidence interval [95% CI] for the difference in accuracy. Diagnostic performance (sensitivity, specificity, and predictive values) for detecting significant CAD (per patient and vessel level) was compared using exact binomial tests and the weighted generalized score methods. To account for the within-patient clustering of vessels, the primary per-vessel analysis was repeated using the modified Obuchowski test for clustered, paired binary data.^25^ Confidence intervals were obtained with a variant of bootstrapping to account for clustering, in which patients were sampled with replacement and vessels were sampled with replacement within each patient. A total of 10 000 clustered samples of vessels were used to obtain confidence intervals. A *P*-value <0.05 was considered statistically significant.

### Sample size

For sample size calculation, we assumed a CAD prevalence of 50% and that both CMR protocols would achieve a diagnostic accuracy of 85%. The 5% non-inferiority margin was considered a clinically acceptable trade-off between diagnostic precision and the potential clinical gains of increased patient throughput and potential cost savings. To show that both tests are equally accurate with a non-inferiority margin of 5% for diagnostic accuracy at the per-vessel level, assuming both protocols disagree no more than 10% of the time, a sample size of 150 participants was required for 90% power (α significance 5%). To allow for missing data in up to 10% of participants, 167 participants were recruited.

## Results

### Subject characteristics

One-hundred and fifty patients with complete CMR and ICA data were included in the final analysis (mean age 66 ± 10 years, 71% male) (*Figure 1*). The median interval between the two scans was 2 [1–3] days, and no significant adverse events occurred during either study. Baseline characteristics are presented in *Table 1*. From angiographic analysis, 43 vessels underwent FFR, and 28 vessels were considered FFR-positive due to the presence of complete or subtotal occlusions. In the remaining vessels, 224 were deemed to require QFR assessment. Overall CAD prevalence was 51% (34 [23%] with single-vessel disease and 42 [28%] with multi-vessel disease). The median interval between the first CMR and ICA was 28 [13–50] days.

**Table 1:** Baseline characteristics

Patients with suspected angina referred for ICA, *n* = 150	
Demographics	
Age, years	66 ± 10
Male	107 (71%)
Body mass index, kg/m^2^	29.8 ± 5.0
Cardiovascular risk factors	
Current smoker	14 (9%)
Ex-smoker	68 (45%)
Hypertension	95 (63%)
Type II diabetes	25 (17%)
Hypercholesterolaemia	70 (47%)
Family history of premature CAD	60 (40%)
Previous myocardial infarction	2 (1%)
Medications	
Aspirin	122 (81%)
P2Y_12_ inhibitor	11 (7%)
Statin	136 (91%)
ACEi/ARB	61 (41%)
Beta blocker	93 (62%)
Calcium channel blocker	54 (36%)
Oral nitrate	59 (39%)
Left ventricular function	
Ejection fraction, %	60.8 ± 9.3
End diastolic volume index, mL/m^2^	76.2 ± 15.6
End systolic volume index, mL/m^2^	30.3 ± 12.1
Mass index, g/m^2^	60.3 ± 13.0
Wall motion score index	1.05 ± 0.16
LGE	
Infarction	24 (16%)
Total infarcted segments	65
Enhanced mass, g^a^	5.98 [3.57–10.27]
Non-ischaemic focal fibrosis	22 (15%)
Invasive coronary angiography	
Functionally significant stenosis	76 (51%)
Single-vessel disease	34 (23%)
Double-vessel disease	26 (17%)
Triple-vessel disease	16 (11%)
LMS disease	6 (4%)
LAD disease	55 (37%)
RCA disease	39 (26%)
LCx disease	36 (24%)
Complete/subtotal occlusion	28 (19%)

Data are presented as mean ± SD, median [Q1–Q3] or absolute value (%).

Abbreviations: ACEi/ARB, angiotensin-converting enzyme inhibitor/angiotensin receptor blocker; CAD, coronary artery disease; LAD, left anterior descending artery; LCx, left circumflex artery; LGE, late gadolinium enhancement; LMS, left main stem; RCA, right coronary artery.

^a^In those with infarction.

### Standard CMR data

CMR functional analysis demonstrated normal mean left ventricular volumes and systolic function, with a mean resting wall motion score index of 1.05 ± 0.16. On qualitative LGE assessment, infarction was evident in 16% of patients, affecting a total of 65 myocardial segments. Non-ischaemic focal fibrosis was present in 15% of patients (*Table 1*).

### Haemodynamic response to adenosine

*Table 2* summarizes the haemodynamic response to adenosine. There were no significant interstudy differences in resting and peak stress heart rate or blood pressure between accelerated and standard protocols. Baseline ECG demonstrated that 97% of participants were in sinus rhythm (four in atrial fibrillation).

**Table 2:** Baseline haemodynamics and response to adenosine

	Standard	Accelerated	*P* value
Baseline			
HR, bpm	63 ± 10	62 ± 10	0.698
SBP, mmHg	139 ± 20	137 ± 20	0.115
DBP, mmHg	78 ± 10	77 ± 11	0.301
Peak stress			
HR, bpm	82 ± 12	81 ± 12	0.563
SBP, mmHg	137 ± 20	135 ± 21	0.234
DBP, mmHg	77 ± 12	76 ± 12	0.111
Change in HR, bpm	19 ± 8	19 ± 8	0.792
Achieved HR ≥10 bpm	133 (89%)	138 (92%)	0.383
Adenosine dose increase	51 (34%)	61 (41%)	0.164

Data are presented as mean ± SD or absolute value (%).

Abbreviations: bpm, beats per minute; DBP, diastolic blood pressure; HR, heart rate; SBP, systolic blood pressure.

### Scan duration

The accelerated CMR protocol was significantly shorter than the standard scan (19 ± 5  vs. 43 ± 8 min respectively, *P* < 0.001) with a mean time saving of 24 min [95% CI: 23, 25].

### Scan tolerability

When comparing patient experience between CMR protocols (questionnaire return rate 86%), 81% of patients rated the accelerated scan as ‘comfortable’, compared to 58% for the standard scan. Furthermore, 59% of participants felt the standard scan was ‘too long’, compared to 4% for the accelerated scan (see Supplementary data online, *Table S1*).

### Image quality

For the standard scan, image quality was rated as ‘excellent’ in 90% of cine imaging, 81% of LGE and 75% of stress perfusion sequences. In comparison, the accelerated scan achieved ‘excellent’ ratings in 85% of cine imaging, 75% of LGE, and 77% of stress perfusion sequences. In both protocols, no sequences were unanalysable (*Table 3*).

**Table 3:** Image quality comparison between standard and accelerated CMR protocols

	Standard	Accelerated
Cine		
Excellent	135 (90%)	127 (85%)
Good	12 (8%)	18 (12%)
Moderate	3 (2%)	5 (3%)
LGE		
Excellent	121 (81%)	112 (75%)
Good	24 (16%)	26 (17%)
Moderate	5 (3%)	12 (8%)
Stress perfusion		
Excellent	112 (75%)	116 (77%)
Good	21 (14%)	26 (17%)
Moderate	17 (11%)	8 (5%)

Data are presented as absolute value (%).

Abbreviation: LGE, late gadolinium enhancement.

### Detection of significant CAD by CMR

#### Per vessel analysis

**Figure 3:**
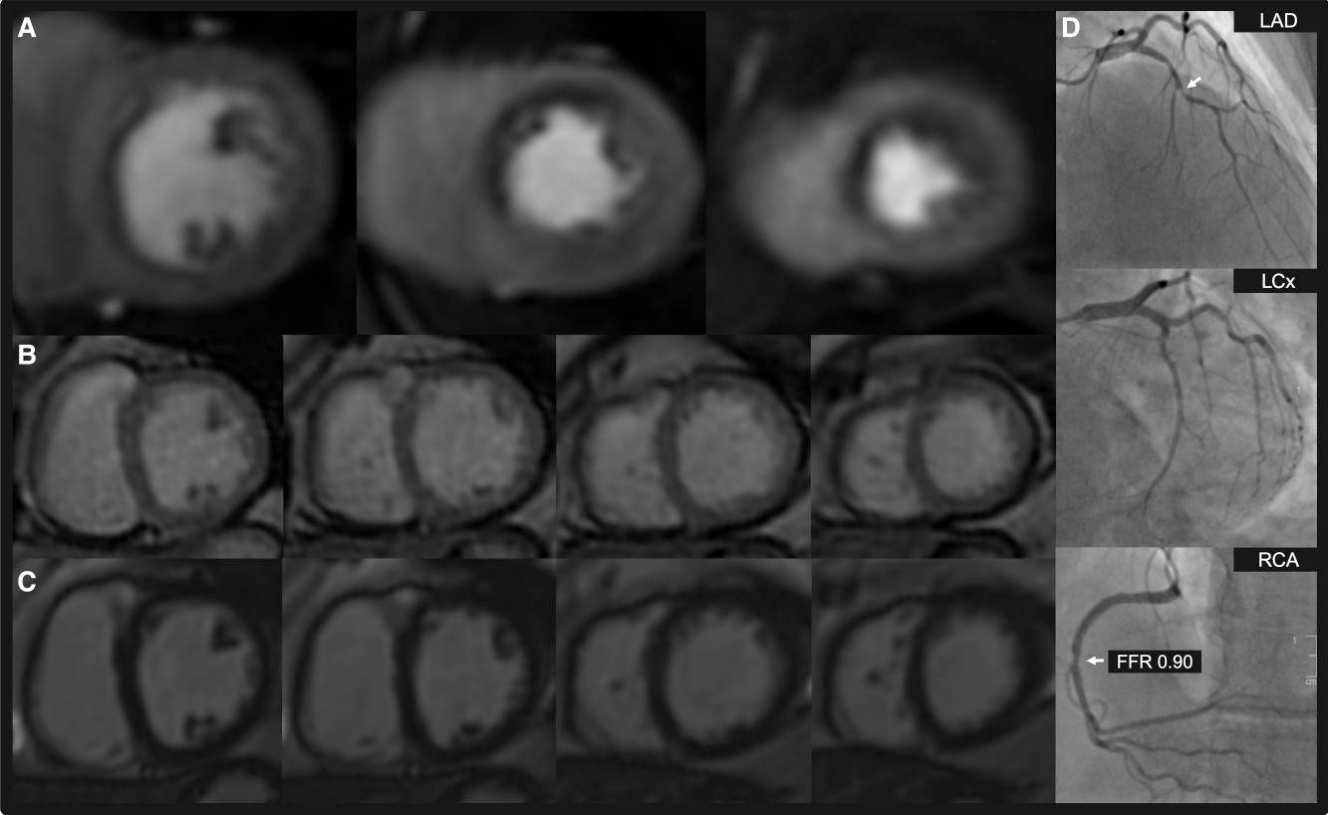
Case example of the accelerated stress-perfusion protocol. An inducible perfusion defect is seen in the LAD territory at all three ventricular levels (*A*) with no accompanying wall motion abnormalities on real-time cine (*B*) or prior infarction on single-shot late gadolinium enhancement (*C*). Invasive coronary angiography confirms a culprit severe LAD stenosis with a moderate non-significant RCA stenosis (*D*). Abbreviations: LAD, left anterior descending artery; LCx, left circumflex artery; RCA, right coronary artery.

**Figure 4:**
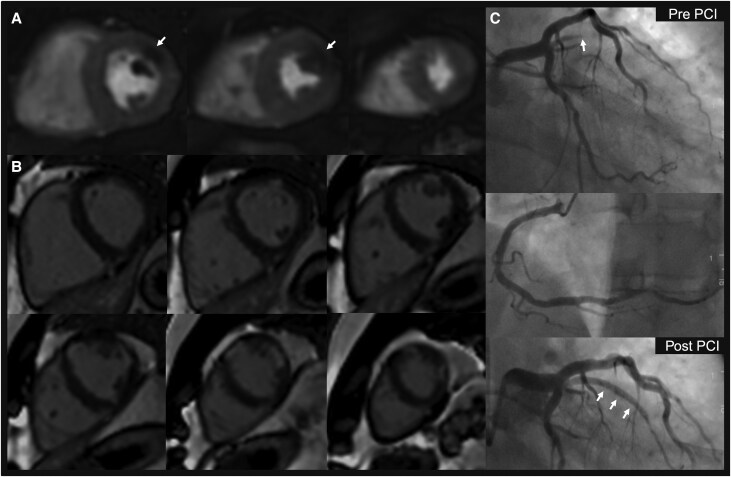
Case example of the accelerated stress–perfusion protocol. Stress perfusion imaging demonstrates a perfusion defect in the basal-mid anterolateral wall (*A*), which mildly exceeds the areas of infarction on single-shot late gadolinium enhancement (*B*). Invasive coronary angiography demonstrates an occluded intermediate branch (*C*). Abbreviations: PCI, percutaneous coronary intervention.

For the primary analysis, with ICA as the reference standard for determining the presence of significant CAD, compared with standard stress CMR, the accelerated protocol achieved non-inferior diagnostic accuracy for reader 1: 82.0% vs. 82.7%, respectively, mean difference +0.7% [95% CI: −2.7%, 4.0%], *p*_non-inferiority_ = 0.001 and reader 2: 82.4% vs. 85.8% respectively, mean difference +3.4% [95% CI: −0.1%, 6.8%], *p*_non-inferiority_ < 0.001 (*Table 4*; *Figures 3 and 4* and Supplementary data online, *Figures S1*–5 for case examples). When accounting for the within-patient clustering of vessels, the accelerated protocol remained non-inferior in diagnostic accuracy for both readers (see Supplementary data online, *Table S2*).

**Table 4:** Diagnostic performance of standard vs. accelerated CMR in detecting significant CAD, with ICA as the reference standard

	Standard CMR	Accelerated CMR	*P* value^a^
**Per vessel**			
*Reader 1*			
Accuracy	82.0% [78.1%, 93.8%]	82.7% [78.8%, 86.1%]	0.791^‡^
Sensitivity	61.2% [52.4%, 69.5%]	59.7% [50.9%, 68.1%]	0.851
Specificity	90.8% [87.1%, 93.8%]	92.4% [88.9%, 95.1%]	0.458
PPV	73.9% [64.7%, 81.8%]	76.9% [67.6%, 84.6%]	0.442
NPV	84.7% [80.4%, 88.3%]	84.4% [80.1%, 88.1%]	0.840
*Reader 2*			
Accuracy	82.4% [78.6%, 85.8%]	85.8% [82.2%, 88.9%]	0.073^§^
Sensitivity	58.2% [49.4%, 66.7%]	68.7% [60.1%, 76.4%]	0.020
Specificity	92.7% [89.3%, 95.3%]	93.0% [89.6%, 95.6%]	1.000
PPV	77.2% [67.8%, 85.0%]	80.7% [72.3%, 87.5%]	0.399
NPV	84.0% [80.0%, 87.6%]	87.5% [83.5%, 90.8%]	0.013
**Per patient**			
Accuracy	84.0% [77.1%, 89.5%]	88.6% [82.4%, 93.3%]	0.189
Sensitivity	76.3% [65.2%, 85.3%]	84.2% [74.0%, 91.6%]	0.180
Specificity	91.9% [83.2%, 97.0%]	93.2% [84.9%, 97.8%]	1.000
PPV	90.6% [80.7%, 96.5%]	92.8% [83.9%, 97.6%]	0.563
NPV	79.1% [69.0%, 87.1%]	85.2% [75.5%, 92.1%]	0.100

Proportions are expressed as percentage [95% confidence interval].

One-sided non-inferiority test with a margin of 5%: ^‡^*P* = 0.001 and ^§^*P* < 0.001.

Abbreviations: NPV, negative predictive value; PPV, positive predictive value.

Significant CAD defined by invasive fractional flow reserve ≤0.80 in epicardial vessels ≥2 mm diameter, or quantitative flow ratio ≤0.80 if FFR not performed.

^a^Two-sided test for difference.

#### Per patient analysis

For secondary analyses, with ICA as the reference standard for determining the presence of significant CAD, for patient-level consensus, standard CMR demonstrated diagnostic accuracy 84.0%, sensitivity 76.3%, and specificity 91.9%. In comparison, accelerated stress CMR achieved diagnostic accuracy 88.6%, sensitivity 84.2%, and specificity 93.2%. When comparing diagnostic accuracy between scans, the accelerated protocol was comparable to standard stress CMR; mean difference 4.6% [95% CI: −1.5%, 11.0%], *P* = 0.189 (*Table 4*). With standard CMR as the reference standard, the accelerated protocol achieved diagnostic accuracy 86.0%, sensitivity 87.5%, and specificity 84.9% (see Supplementary data online, *Table S3*).

#### Interobserver variability

For qualitative analysis, interobserver agreement at the patient level was excellent for both the standard and accelerated CMR protocols (Kappa coefficient 0.85 [95% CI: 0.76, 0.94] and 0.88 [95% CI: 0.80, 0.96], respectively) (*Table 5*). At the vessel level, interobserver agreement was moderate for both the standard and accelerated CMR protocols (*Table 5*). In one case, consensus was not reached and was resolved by the third adjudicator.

**Table 5:** Interobserver agreement on the presence of significant CAD

	Standard CMR	Accelerated CMR
**Per patient**		
Kappa coefficient	0.85 [0.76, 0.94]	0.88 [0.80, 0.96]
**Per vessel**		
Kappa coefficient	0.70 [0.63, 0.78]	0.72 [0.65, 0.80]

Proportions are expressed as absolute value [95% confidence interval].

## Discussion

In this prospective, powered diagnostic accuracy trial examining the non-invasive detection of significant CAD by CMR, we demonstrate that an accelerated stress–only perfusion protocol achieves non-inferior accuracy compared to standard stress–rest perfusion CMR and can shorten the average scan duration by 24 min. To our knowledge, this represents the first prospective, randomized, paired trial of accelerated stress–perfusion CMR with an invasive reference standard. Our findings show that an accelerated protocol can maintain excellent diagnostic accuracy in patients with suspected CAD, consistent with the performance attained by standard stress–perfusion CMR in the published literature (pooled sensitivity 84% and specificity 85%),^8^ but can also dramatically reduce scan times and improve patient tolerability.^13,14^ Thus, accelerated imaging has the potential to improve the accessibility, efficiency and cost-effectiveness of CMR and enhance patient care through a more streamlined examination.

Previous studies have demonstrated the feasibility of accelerated protocols but did not examine diagnostic performance. In a small study of 18 patients with suspected or known CAD, combined functional, ischaemia and viability assessment was completed in 17.2 ± 0.5 min.^13^ In another study, patients underwent either a standard (*n* = 80, average scan duration 36 min [range 24–52]) or accelerated (*n* = 120, average scan duration 23 min [range 14–31]) stress protocol, resulting in a time saving of 13 min and facilitating service delivery (an additional three scans per day per scanner).^14^ A multicentre, prospective trial involving 601 patents in five low-to-medium income countries demonstrated the cost-effectiveness of rapid non-stress CMR protocols for cardiomyopathy detection. Average scan duration was 22 ± 6 min for contrast-enhanced scans and 12 ± 4 min for non-contrast, with diagnostic imaging achieved in 98% and cost reductions between 30 and 60%.^26^

Stress–perfusion CMR is recommended as the first-line imaging strategy for patients with known CAD or a moderate-high risk of disease.^7,27^ In the UK, nearly half of clinical CMR referrals necessitate ischaemia or viability assessment.^11^ However, the sharp rise in demand for CMR over the past decade, coupled with limited and regionally disparate numbers of scanners and specialist reporters, restricts equitable access.^11,12^ In the USA, inconsistent reimbursement policies and stringent pre-authorization requirements further hinder widespread adoption.^28^ These inequalities directly affect patient care, delaying diagnoses due to long waiting lists and forcing reliance on less accurate tests or direct referrals for ICA, which has a low diagnostic yield when utilized first-line.^29^ Hence, enhancing the efficiency of existing CMR protocols is essential to streamline services and meet rising demand.

Integrating accelerated imaging protocols into clinical practice may address these barriers through: (i) shorter scan times—to boost patient throughput, reduce waiting lists and improve accessibility; (ii) reduced costs from shorter scans and reduced contrast use (one-third less through omission of rest perfusion); and (iii) enhanced patient comfort and tolerability from shorter, free-breathing scan protocols. In our study, the accelerated protocol averaged 19 min, potentially enabling booking slots to be reduced from 60 to 40 min, thus allowing increased patient throughput.

Previous studies have proven CMR as a cost-effective diagnostic strategy in stable CAD. The European CMR registry showed significant cost savings with an upfront CMR strategy to target ICA referrals in three European public sectors (with cost savings of 23–50%).^30^ In a CE-MARC (Clinical Evaluation of Magnetic Resonance Imaging in Coronary Heart Disease) substudy analysis evaluating eight diagnostic strategies for CAD, involving combinations of exercise treadmill testing, single-photon emission computed tomography, CMR and ICA, only two strategies, both including CMR, emerged as potentially cost-effective.^31^ A subsequent economic evaluation of CE-MARC 2 confirmed the clinical utility of CMR, with higher estimated quality-adjusted life years gain while incurring costs comparable to those of myocardial perfusion scintigraphy and National Institute for Health and Care Excellence guideline-directed care.^32^ Furthermore, real-world data in 2298 patients showed increased costs of alternative functional modalities such as computed tomography coronary angiography-derived FFR compared with stress–perfusion CMR.^33^

A typical stress CMR protocol includes cine imaging for functional and wall motion assessment, LGE to detect prior infarction and perfusion imaging for ischaemia assessment.^16^ This approach affords high diagnostic performance to detect significant CAD (accuracy 84%, sensitivity 82%, and specificity 86%).^34^ However, perfusion imaging has the highest individual diagnostic performance to detect significant CAD (accuracy 86%, sensitivity 77%, and specificity 92%), with LGE and ventricular functional analysis reporting a high specificity (both >90%) but lower sensitivity in isolation (41 and 47%, respectively).^34^ Therefore, there is potential to accelerate the LGE and functional components of a stress protocol whilst maintaining diagnostic confidence for a reliable CAD assessment. This combined approach is valuable not only for its high diagnostic yield but also for the incremental prognostic value in patients with CAD, aiding decisions regarding revascularization and informing potential targeted drug or device therapies.^35–38^

As demonstrated in our study, omitting rest perfusion did not result in a reduction in accuracy when compared with a standard stress–perfusion protocol. These findings add to the growing body of evidence that rest perfusion provides limited additional diagnostic value, and as such, can be removed from stress protocols to shorten examination times.^39^ A CE-MARC substudy retrospectively analysed 666 patients, demonstrating that a stress-LGE approach had superior diagnostic accuracy in detecting significant CAD compared with a conventional stress–rest assessment (area under the curve 0.843 vs. 0.834, respectively, *P* = 0.02).^40^ In another retrospective study, although inclusion of rest perfusion improved clinician confidence in detecting significant CAD, it did not improve diagnostic performance across readers with varying levels of experience.^41^ However, to our knowledge, our study is the first to assess a stress–LGE approach prospectively using an invasive reference standard, performed in a randomized order and therefore mitigating ascertainment bias. Moreover, LGE imaging is superior to rest perfusion in detecting infarction,^42^ and hence, omitting rest perfusion could save time and expense without compromising diagnostic performance. However, this approach would forgo the ability to determine myocardial perfusion reserve, which has been shown to independently predict adverse cardiovascular outcomes.^43^ Nevertheless, the diagnostic yield of myocardial perfusion reserve has not been shown to surpass that of hyperaemic myocardial blood flow.^44^

With a composite time saving of 24 min, primarily driven by acceleration of cine and LGE imaging (each with a typical duration of approximately 90 s in our institution), and to a lesser extent, omission of rest perfusion, these efficiencies must be weighed against the potential diagnostic trade-offs. Cine imaging remains an integral component of a stress-perfusion protocol,^16^ but offers limited additional diagnostic value beyond perfusion and LGE imaging, supporting its acceleration to maintain overall protocol efficiency. The use of free-breathing, single-shot LGE and real-time cine eliminates the need for sequential breath-holds and multiple segmented acquisitions, substantially reducing total scan time and patient burden. However, this may compromise image quality, scar detection, and reproducibility. Although real-time cine has shown good agreement with standard cine imaging for volumetric and wall motion assessment,^19^ the low infarct prevalence in this study precluded a meaningful reproducibility assessment for single-shot LGE imaging. Furthermore, LGE contrast may vary due to differences in total contrast dose (0.1 mmol/kg for accelerated CMR vs. 0.15 mmol/kg for standard CMR) and, given the accelerated nature of single-shot LGE, scar visibility may be affected by timing differences between contrast administration and image acquisition. Another consideration is that the omission of rest perfusion, whilst reducing scan time and contrast use, may impact the ability of less experienced readers to adjudicate artefacts.

## Study limitations

This was a single-centre study, which was not powered at the patient level, and patients with prior percutaneous coronary intervention or coronary artery bypass grafting were excluded. Thus, further validation in a larger, multicentre trial evaluating performance in broader clinical populations is required before clinical implementation can be recommended and its potential impact on patient outcomes assessed. Despite robust blinding procedures for CMR and ICA image analysis, FFR assessment was performed at the discretion of the clinical team and, hence, was not available in all cases. As such, wire-free functional assessment with QFR computation was performed in the remaining vessels, which has shown good agreement with invasively determined FFR.^45^ The presence of coronary microvascular dysfunction was not determined invasively and may have resulted in an underestimation of FFR and discordance with false positive stress–perfusion reads. Although quantitative CMR perfusion may provide greater objectivity in determining the severity and extent of ischaemia, it remains primarily a research tool, currently with limited model-, vendor-, and field strength-specific data. For this reason, we performed a qualitative read to ensure that our findings are readily translatable into routine clinical practice. Moreover, as the study was designed to evaluate the utility of a clinically applicable protocol, individual module-specific accuracy was not evaluated. Finally, studies were performed at 3-Tesla, and hence we did not assess performance at 1.5 Tesla, which is more commercially available; thus, our findings may have limited wider generalizability to lower field-strength systems. However, the purpose of our study was to provide a robust head–head diagnostic comparison and determine whether accelerated protocols have a role in the clinical arena without compromising diagnostic performance.

## Conclusions

In this prospective randomized study, an accelerated stress–perfusion CMR protocol achieved non-inferior diagnostic accuracy compared with standard stress–perfusion CMR for the detection of significant CAD at the vessel level. This streamlined workflow dramatically reduces scan duration and enhances patient tolerability. Further research is warranted to explore the long-term impact of incorporating accelerated CMR into routine clinical practice.

## Supplementary Material

jeaf322_Supplementary_Data

## Data Availability

Data underlying this article will be shared on reasonable request to the corresponding author.
