# Supplementary material for: Accelerated stress CMR for the detection of significant coronary artery disease: a prospective randomized diagnostic accuracy study
Source: Eur Heart J Cardiovasc Imaging. 2025 Nov 20;27(4):597–607. doi: 10.1093/ehjci/jeaf322 (PMC13021280; doi:10.1093/ehjci/jeaf322)
Supplement: jeaf322_Supplementary_Data [file jeaf322_supplementary_data.zip › Accelerated_CMR_Supplementary.docx]

**Supplementary Files**

**Supplementary Table 1:** Comparison of patient-reported experience between the standard and accelerated CMR protocols

|  | **Standard, n=123** | | **Accelerated, n=116** |
| --- | --- | --- | --- |
| **Overall scan experience** | |  |  |
| Comfortable | | 72 (58%) | 94 (81%) |
| Uncomfortable | | 43 (35%) | 21 (18%) |
| Very uncomfortable | | 8 (7%) | 1 (1%) |
| **Perceived scan duration*** | |  |  |
| Shorter than expected | | 8 (7%) | 86 (75%) |
| About right | | 42 (34%) | 25 (22%) |
| Too long | | 63 (51%) | 3 (3%) |
| Far too long | | 10 (8%) | 1 (1%) |
| **Symptoms experienced with adenosine**** | |  |  |
| Comfortable | | 32 (26%) | 64 (55%) |
| Uncomfortable | | 84 (69%) | 48 (41%) |
| Very uncomfortable | | 6 (5%) | 4 (3%) |
| **Recommend scan to a family member** | | 123 (100%) | 115 (100%) |

Data presented as absolute value (%)

*Accelerated survey n=115, **standard survey n=122

**Supplementary Table 2:** Diagnostic performance of standard vs. accelerated CMR in detecting significant CAD (per-vessel, adjusted for clustering), with invasive coronary angiography as the reference standard

|  | **Standard CMR** | **Accelerated CMR** | **P value*** |
| --- | --- | --- | --- |
| **Per vessel** |  |  |  |
| ***Reader 1*** |  |  |  |
| Diagnostic accuracy | 82.0% [77.8%, 86.2%] | 82.7% [78.7%, 86.7%] | 0.715 |
| Sensitivity | 61.2% [52.0%, 70.3%] | 59.7% [50.8%, 68.4%] | 0.706 |
| Specificity | 90.8% [86.7%, 94.5%] | 92.4% [88.8%, 95.7%] | 0.466 |
| PPV | 73.9% [64.3%, 83.2%] | 76.9% [67.7%, 85.7%] | 0.637 |
| NPV | 84.7% [80.0%, 89.1%] | 84.4% [79.9%, 88.6%] | 0.864 |
| ***Reader 2*** |  |  |  |
| Diagnostic accuracy | 82.4% [78.2%, 86.4%] | 85.8% [82.0%, 89.3%] | 0.083 |
| Sensitivity | 58.2% [48.5%, 67.7%] | 68.7% [59.7%, 77.1%] | 0.040 |
| Specificity | 92.7% [88.9%, 96.1%] | 93.0% [89.3%, 96.3%] | 0.887 |
| PPV | 77.2% [66.9%, 86.9%] | 80.7% [72.0%, 88.9%] | 0.599 |
| NPV | 84.0% [79.3%, 88.3%] | 87.5% [83.3%, 91.3%] | 0.465 |

*Two-sided test for difference

***Reader 1:*** Difference in accuracy +0.7% [-2.9%, +4.2%], p<0.001 (one-sided non-inferiority at 5%)

***Reader 2:*** Difference in accuracy +3.4% [-0.4%, +7.1%], p<0.001 (one-sided non-inferiority at 5%)

**Supplementary Table 3:** Diagnostic performance of accelerated CMR in detecting significant CAD with standard CMR as the reference standard

|  | **Accelerated CMR** | **[95% CI]** |
| --- | --- | --- |
| **Per patient** |  |  |
| Diagnostic accuracy | 86.0% | [79.4%, 91.1%] |
| Sensitivity | 87.5% | [76.8%, 94.4%] |
| Specificity | 84.9% | [75.5%, 91.7%] |
| PPV | 81.2% | [69.9%, 89.6%] |
| NPV | 90.1% | [81.5%, 95.6%] |

Proportions expressed as percentage [95% confidence interval]

**Abbreviations:** CAD coronary artery disease, CMR cardiovascular magnetic resonance, NPV negative predictive value, PPV positive predictive value

**Supplementary Figures**

**Supplementary Figure 1**

**
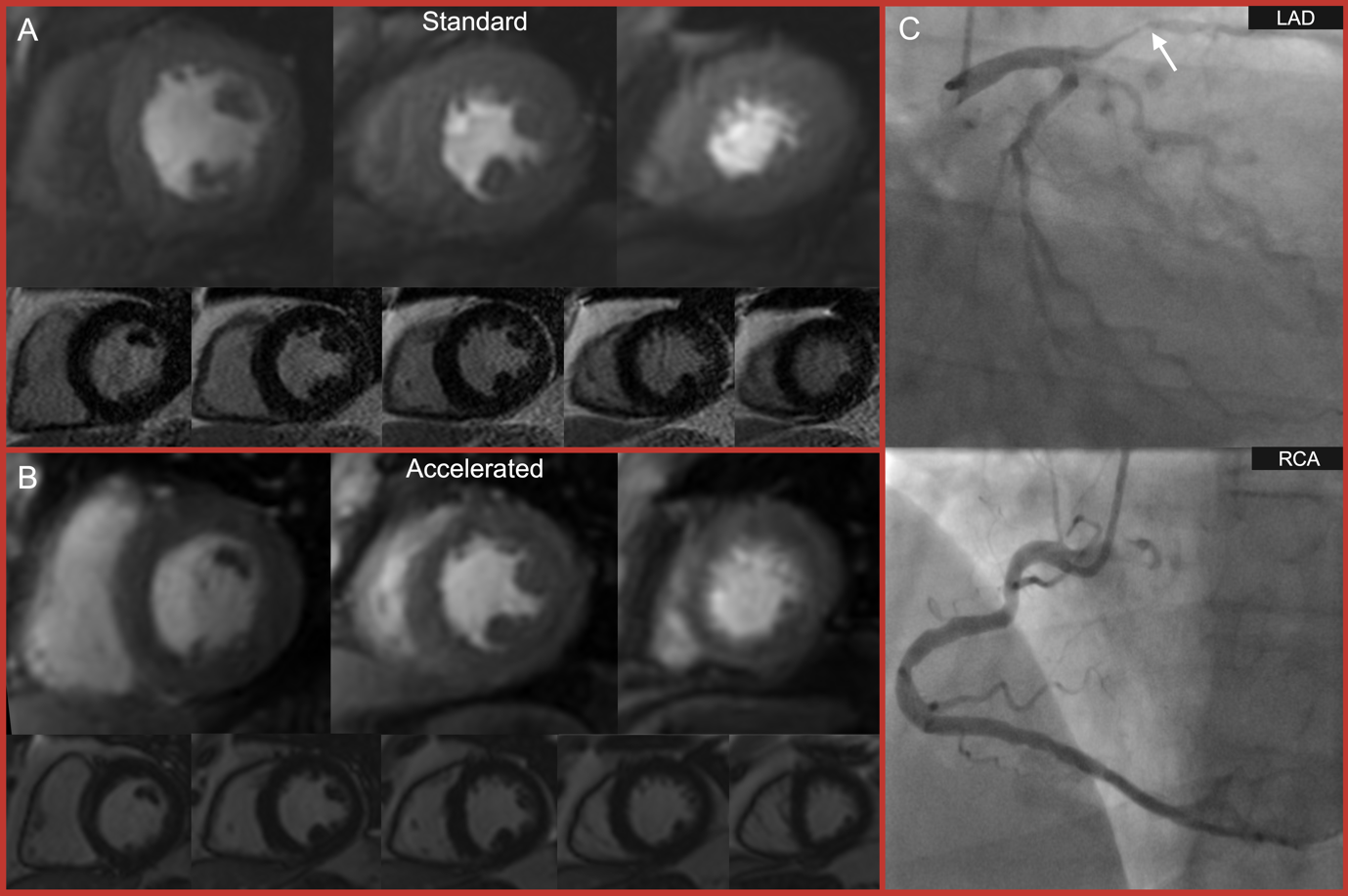
**

Both the standard (panel A) and accelerated (panel B) CMR protocols demonstrate no evidence of an inducible perfusion defect or infarction. However, both protocols miss the presence of significant CAD with invasive coronary angiography (panel C) demonstrating a severe proximal stenosis of the left anterior descending artery.

**Supplementary Figure 2**


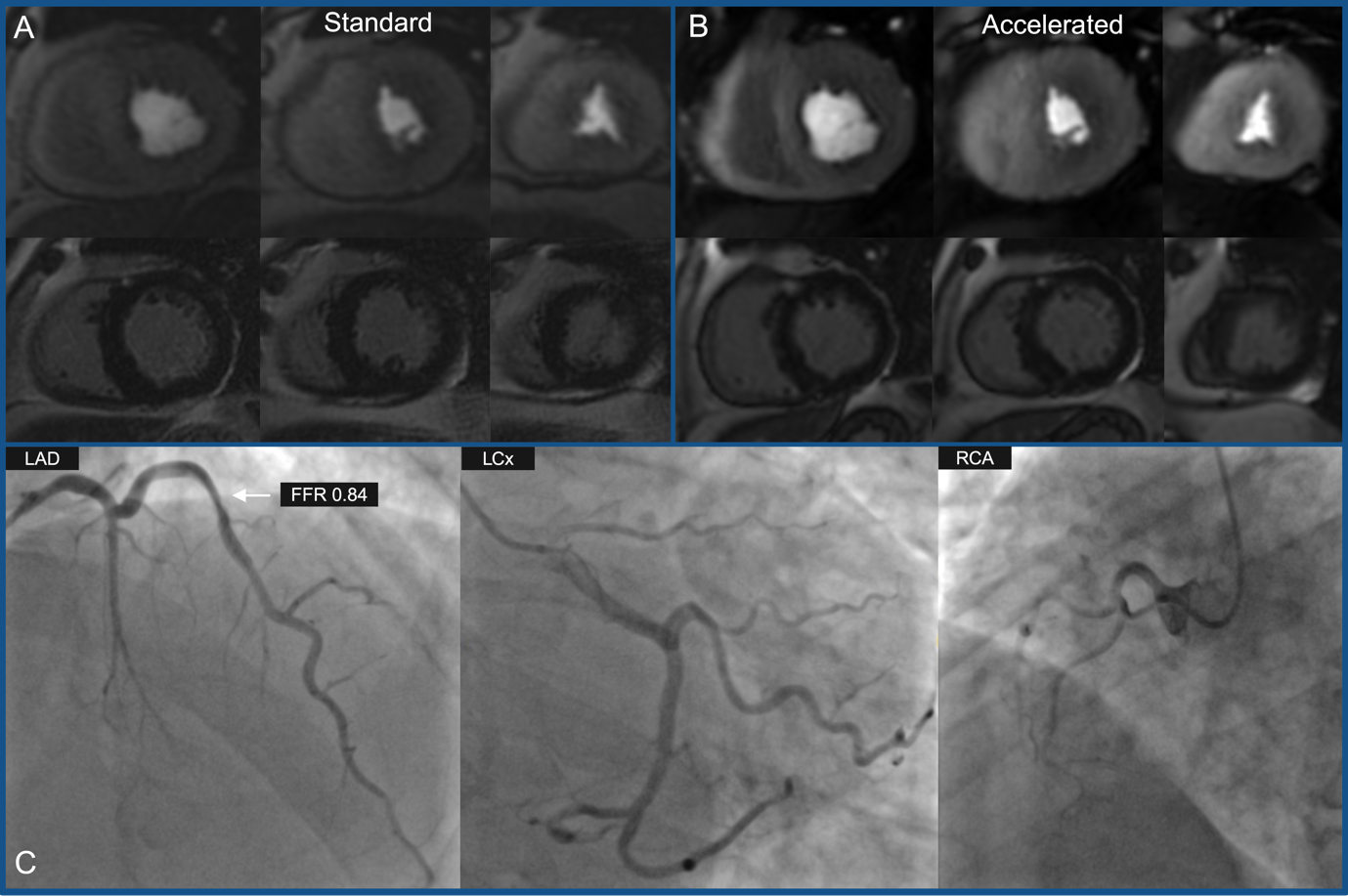


Both the standard (panel A) and accelerated (panel B) CMR protocols demonstrate a stress-induced circumferential perfusion defect at all three ventricular levels. The standard scan was interpreted as three-vessel disease whereas the accelerated scan was deemed to show coronary microvascular dysfunction. Invasive coronary angiography (panel C) demonstrated normal epicardial coronary arteries.

**
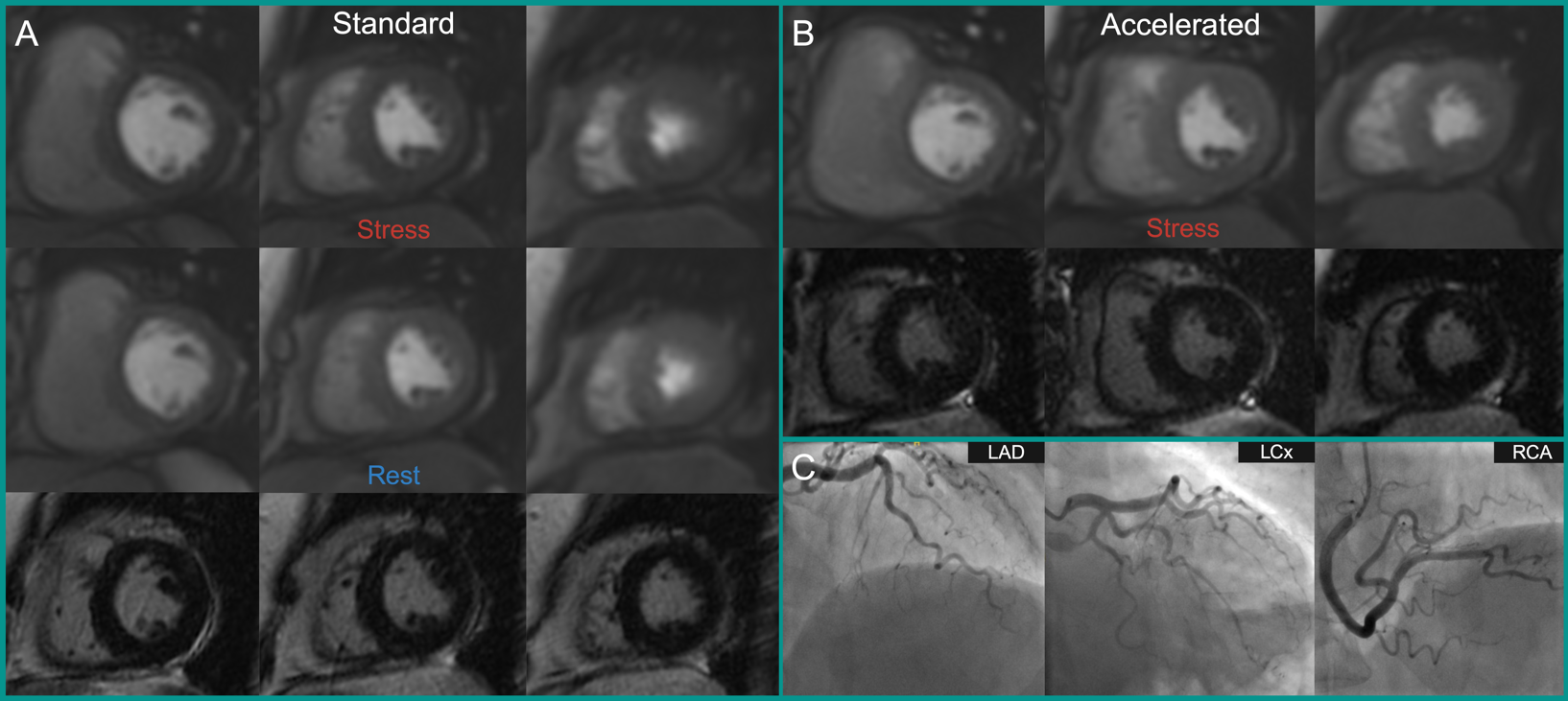
Supplementary Figure 3**

No evidence of a stress-induced perfusion defect or infarction on the standard (panel A) or accelerated (panel B) CMR scans. Omission of rest perfusion resulted in no reduction in diagnostic confidence on the presence of dark rim artefact appreciated in both CMR studies. Invasive coronary angiography (panel C) confirmed the presence of normal epicardial coronary arteries.

**Supplementary Figure 4**

**
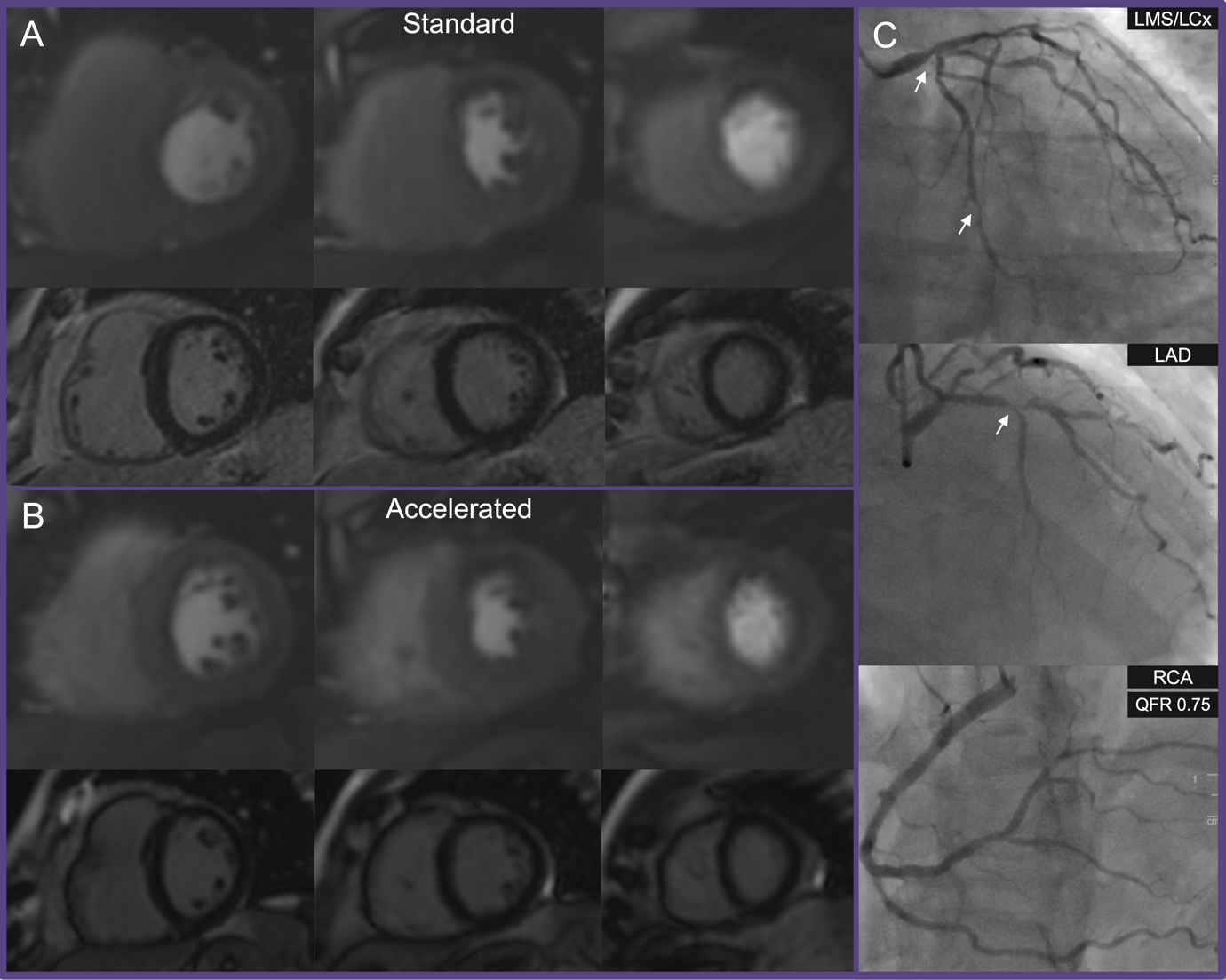
**

Both the standard (panel A) and accelerated (panel B) CMR protocols demonstrate a stress-induced perfusion defect in the left anterior descending territory. However, CAD extent was underestimated in both studies with invasive coronary angiography (panel C) demonstrating significant three-vessel CAD.

**
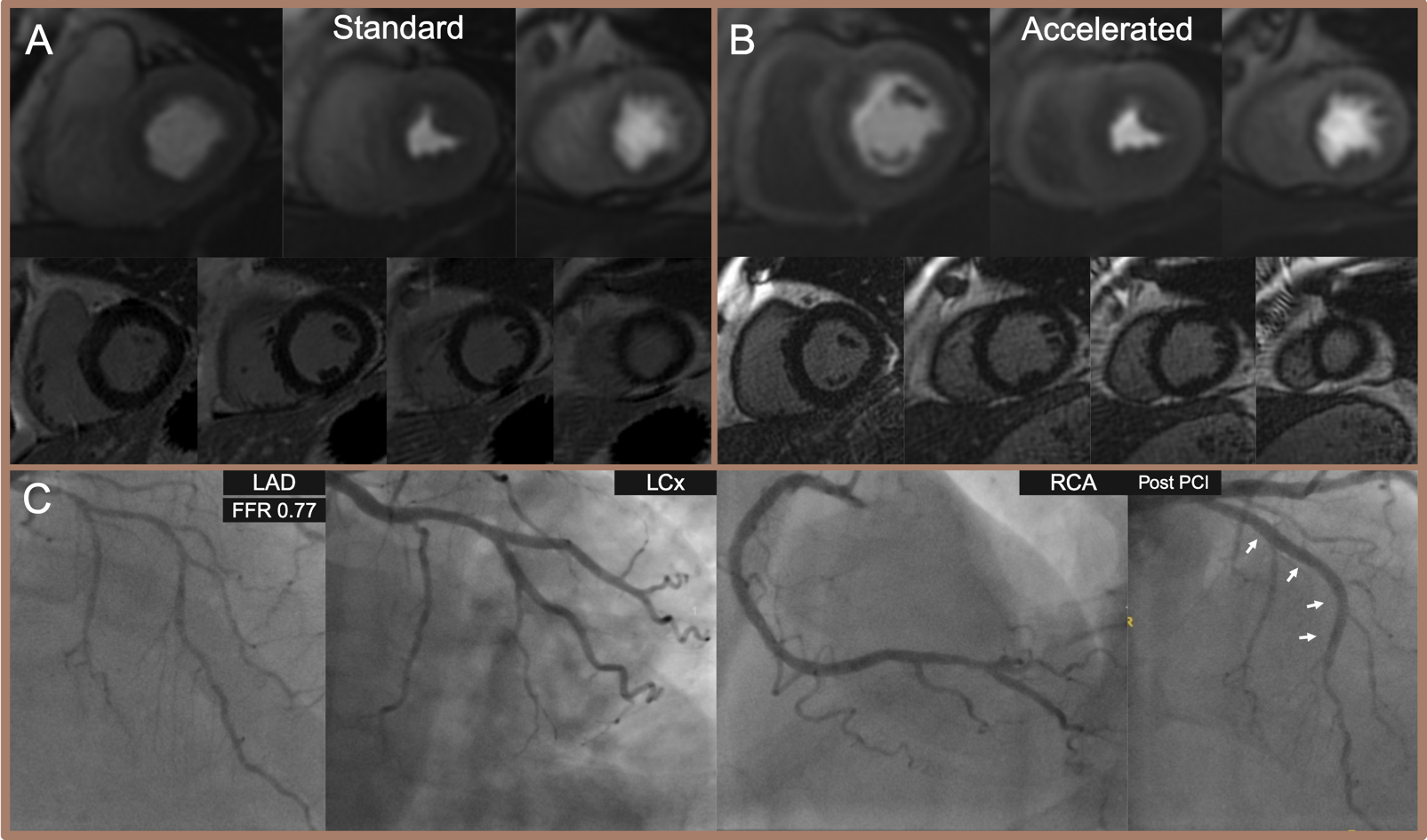
Supplementary Figure 5**

The standard (panel A) and accelerated (panel B) CMR protocols demonstrate a stress-induced circumferential perfusion defect at all three ventricular levels, both interpreted as showing three-vessel CAD. However, invasive coronary angiography (panel C) demonstrated single-vessel disease with significant stenoses of the left anterior descending artery requiring percutaneous coronary intervention.
