# Supplementary figures and images for: Accelerated stress CMR for the detection of significant coronary artery disease: a prospective randomized diagnostic accuracy study
Source: Eur Heart J Cardiovasc Imaging. 2025 Nov 20;27(4):597–607. doi: 10.1093/ehjci/jeaf322 (PMC13021280; doi:10.1093/ehjci/jeaf322)

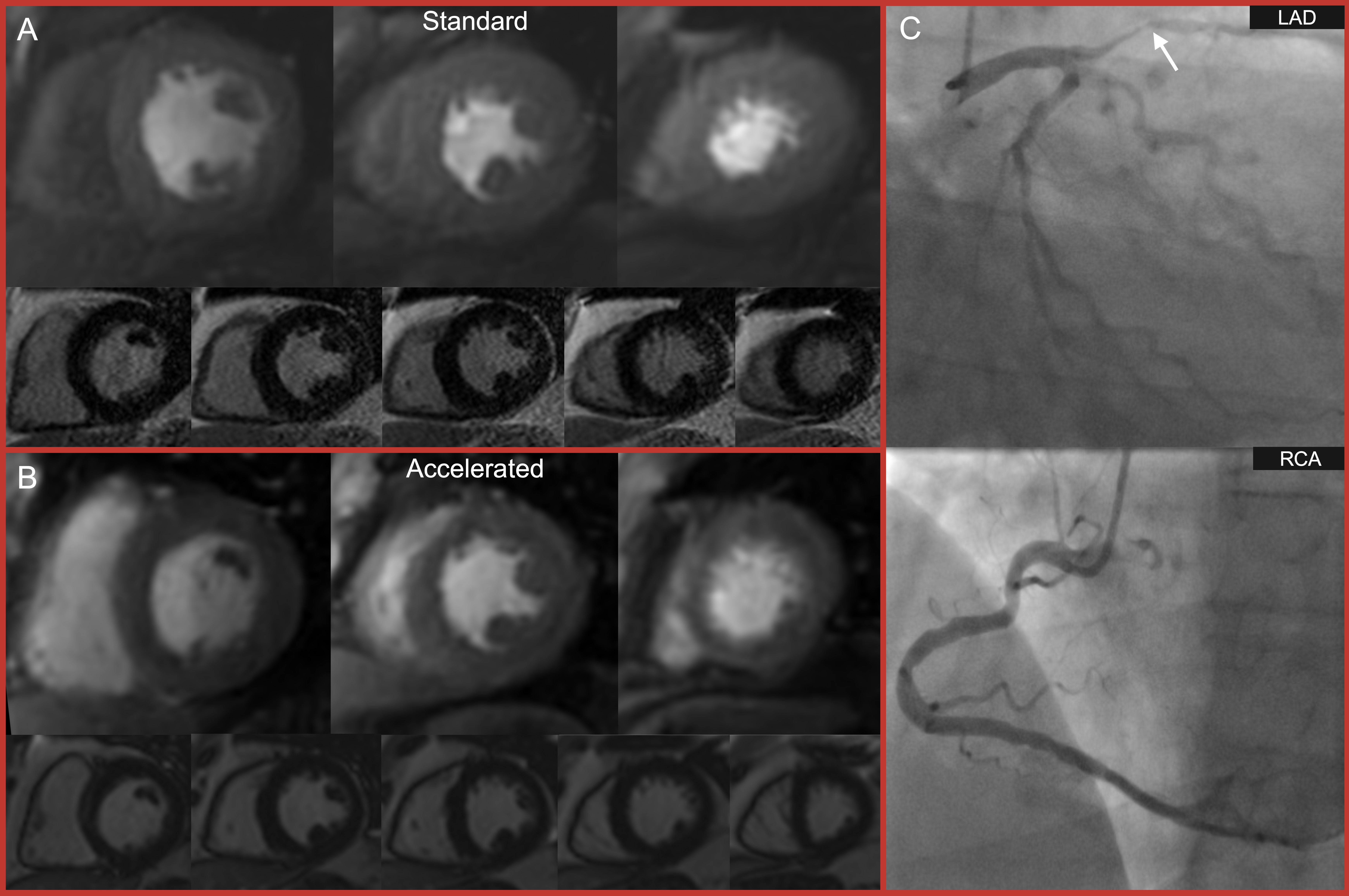

Supplement: jeaf322_Supplementary_Data [file jeaf322_supplementary_data.zip › Supplementary Figure 1.png]

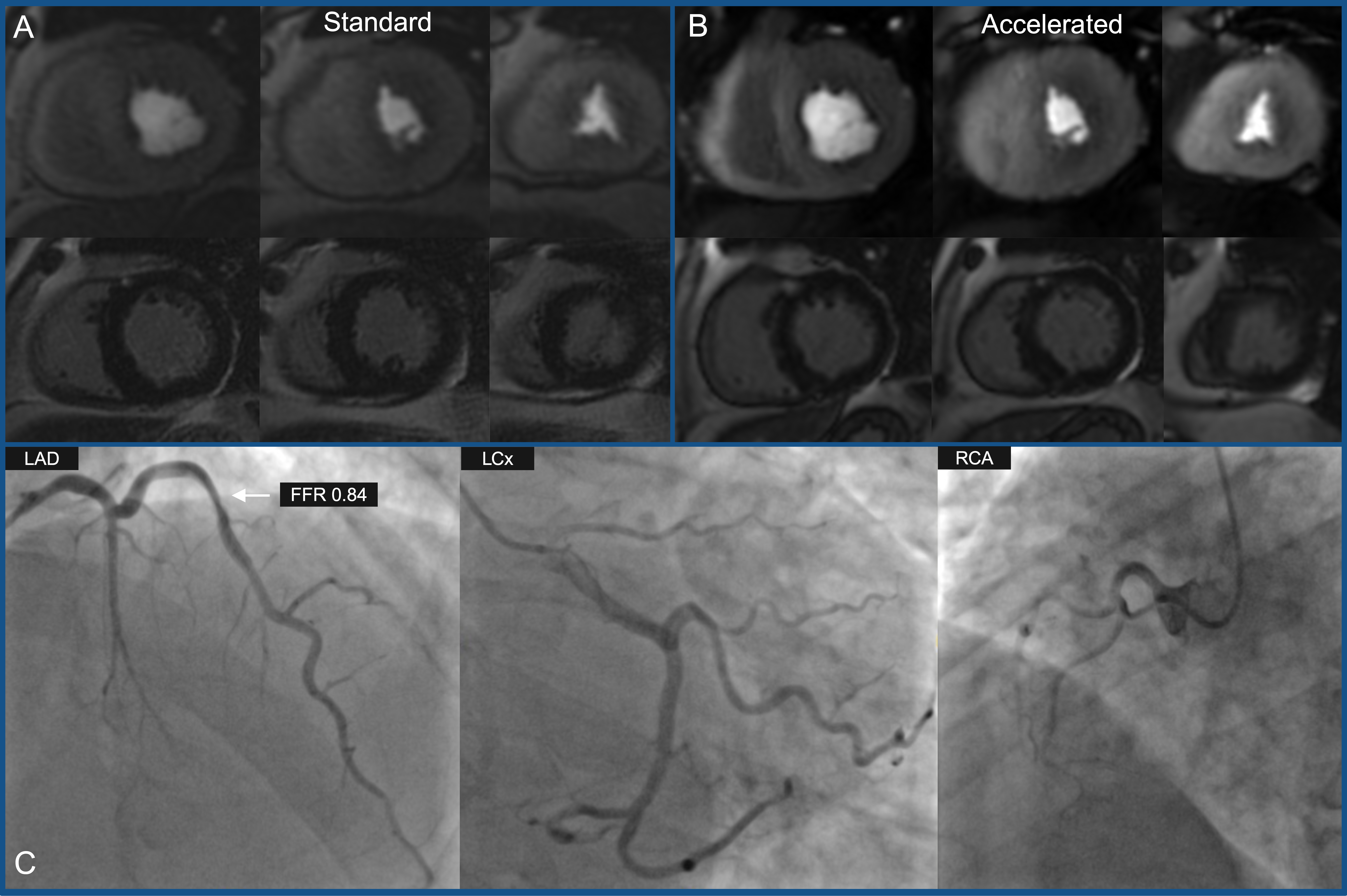

Supplement: jeaf322_Supplementary_Data [file jeaf322_supplementary_data.zip › Supplementary Figure 2.png]

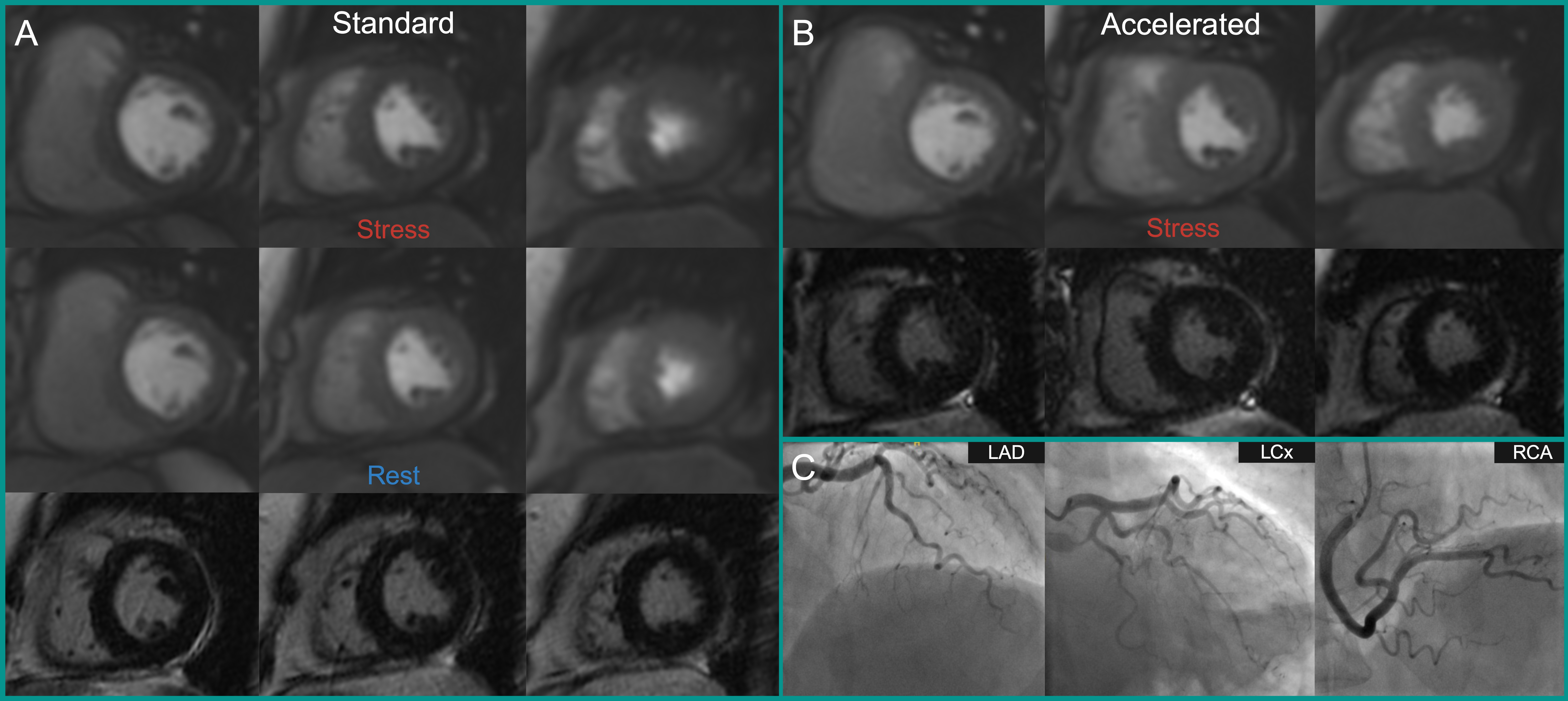

Supplement: jeaf322_Supplementary_Data [file jeaf322_supplementary_data.zip › Supplementary Figure 3.png]

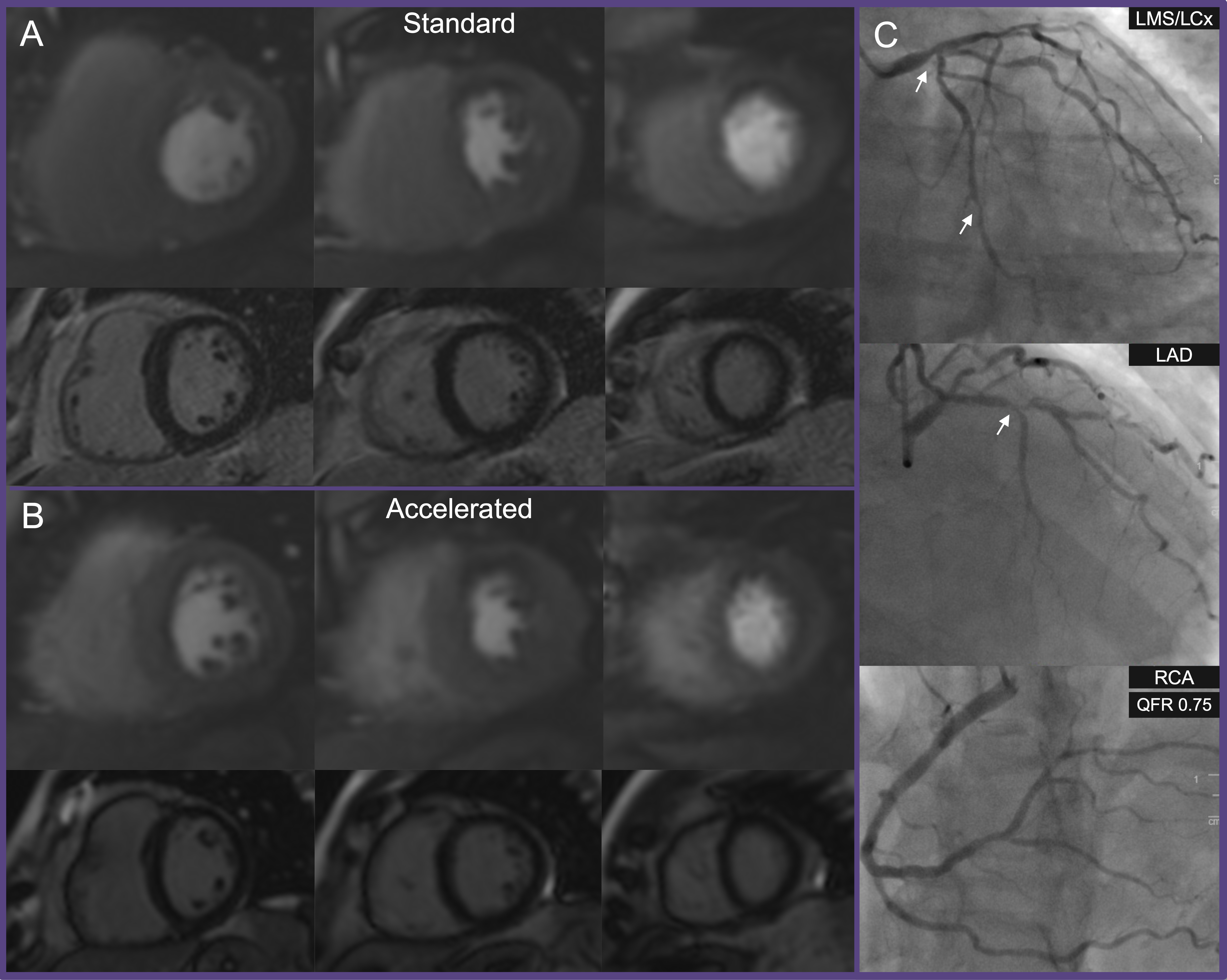

Supplement: jeaf322_Supplementary_Data [file jeaf322_supplementary_data.zip › Supplementary Figure 4.png]

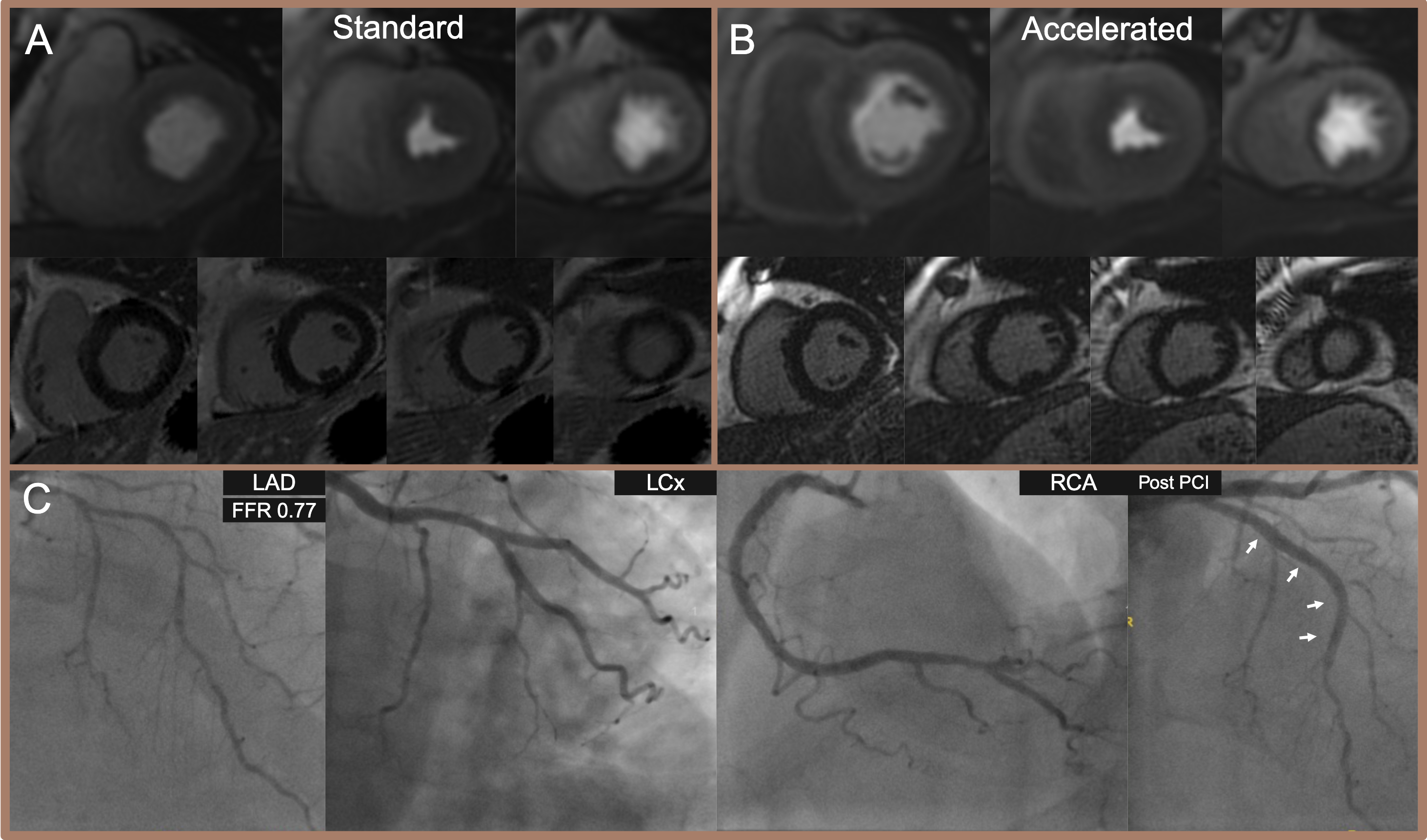

Supplement: jeaf322_Supplementary_Data [file jeaf322_supplementary_data.zip › Supplementary Figure 5.png]
